# Towards Safe and Effective Biomedical Nanocoatings: Plasma-Sputtered Magnesium-Based Nanoparticles with Cytoprotective, Antimicrobial and Antialgal Properties

**DOI:** 10.3390/molecules30173526

**Published:** 2025-08-28

**Authors:** Raminta Rodaitė, Laura Kairytė, Agnė Giedraitienė, Modestas Ružauskas, Rita Šiugždinienė, Ieva Čiapienė, Vacis Tatarūnas, Šarūnas Varnagiris, Darius Milčius

**Affiliations:** 1 Center for Hydrogen Energy Technologies, Lithuanian Energy Institute, Breslaujos Str. 3, LT-44403 Kaunas, Lithuania; kairytelaura2000@gmail.com (L.K.); sarunas.varnagiris@lei.lt (Š.V.); darius.milcius@lei.lt (D.M.); 2 Faculty of Natural Sciences, Vytautas Magnus University, Universiteto Str. 10, LT-53361 Akademija, Lithuania; 3 Institute of Microbiology and Virology, Faculty of Veterinary Medicine, Lithuanian University of Health Sciences, Mickeviciaus Str. 9, LT-44307 Kaunas, Lithuania; agne.giedraitiene@lsmu.lt (A.G.); modestas.ruzauskas@lsmu.lt (M.R.); rita.siugzdiniene@lsmu.lt (R.Š.); 4 Institute of Cardiology, Lithuanian University of Health Sciences, Sukileliu 15, LT-50103 Kaunas, Lithuania; ieva.ciapiene@lsmu.lt (I.Č.); vacis.tatarunas@lsmu.lt (V.T.)

**Keywords:** magnetron sputtering, magnesium nanoparticles, antimicrobial coatings, cytocompatibility, eco-friendly nanotechnology, biomedical textiles

## Abstract

The demand for antimicrobial and biocompatible materials in biomedical applications continues to grow, particularly in the context of wound care and textiles. This study explores the development of multifunctional coatings by applying magnesium (Mg) nanoparticles onto medical-grade cotton textiles using magnetron sputtering—a solvent-free and environmentally sustainable technique. A comprehensive material characterization confirmed the formation of Mg, MgO and Mg(OH)_2_/MgH_2_ phases, along with generally consistent particle coverage and increased fiber surface roughness. The antibacterial testing revealed the effective inhibition of both Gram-positive and Gram-negative bacteria—except *Enterococcus faecalis.* Additionally, the growth of the fungus *Candida albicans* and the microalgae *Prototheca* spp. was reduced by over 80%. Importantly, a cytocompatibility evaluation using human umbilical vein endothelial cells (HUVECs) demonstrated not only non-toxicity but a significant increase in cell viability after 72 h, particularly in samples treated for 20 and 60 min, indicating a potential cytoprotective and proliferative effect. These findings highlight the dual functionality of plasma-sputtered Mg nanoparticle coatings, offering a promising strategy for the development of eco-friendly, antimicrobial and cell-supportive medical textiles.

## 1. Introduction

The growing demand for multifunctional, non-cytotoxic and environmentally sustainable antimicrobial materials for biomedical textiles has intensified research in applications such as wound healing, tissue engineering and infection prevention [1,2,3,4]. Traditional antimicrobial coatings often use silver or copper nanoparticles, which, despite their effectiveness, raise concerns due to cytotoxicity and long-term biosafety issues [5,6]. Consequently, increasing attention has been given to alternative antimicrobial agents that combine broad-spectrum efficacy with low cytotoxicity towards human cells. These include nanosized materials such as titanium dioxide (TiO_2_), zinc oxide (ZnO), copper oxide (CuO) and gold (Au) nanoparticles [7], as well as non-nanosized agents such as chitosan, quaternary ammonium compounds and plant-derived bioactives [8,9,10]. While these alternatives offer benefits such as biocompatibility, biodegradability and a reduced risk of adverse effects, they may also face limitations including photocatalytic dependency (TiO_2_), high production costs (Au nanoparticles) or limited durability under repeated washing and environmental exposure (e.g., chitosan-based coatings) [8,11,12]. Meanwhile, magnesium (Mg), an essential trace element in the human body, is gaining considerable attention owing to its intrinsic antibacterial properties, physiological relevance and favorable biocompatibility profile [13,14]. The release of Mg^2+^ ions is essential for a variety of biological functions, including the activation of key enzymes, cellular energy production and neuromuscular regulation [15,16]. Magnesium plays an important role in cellular proliferation, adhesion and migration, especially in vascular and epithelial cells [17].

At the mitochondrial level, Mg^2+^ acts as a cofactor for critical enzymes such as isocitrate dehydrogenase, pyruvate dehydrogenase and α-ketoglutarate dehydrogenase, all of which are integral to the tricarboxylic acid (TCA) cycle. By enhancing ATP synthesis and regulating calcium dynamics, magnesium supports cellular energy homeostasis and reduces apoptosis under physiological conditions [16]. Numerous studies have shown that Mg^2+^ promotes the proliferation of endothelial cells, including human umbilical vein endothelial cells (HUVECs). Physiological Mg^2+^ concentrations (~1.0–1.5 mM) support normal endothelial function, while concentrations in the range of 2.0–5.0 mM further enhance proliferation and facilitate wound healing, particularly in oxidative or hyperglycemic environments [18,19]. Conversely, magnesium deficiency (<0.3 mM) has been shown to impair cell viability and increase oxidative stress.

Among Mg-based compounds, magnesium oxide (MgO), magnesium hydroxide (Mg (OH)_2_) and magnesium hydride (MgH_2_) all exhibit notable antimicrobial properties. MgO and Mg (OH)_2_ act by generating reactive oxygen species (ROS), disrupting bacterial membranes and increasing local pH, effectively inhibiting the growth of Gram-positive and Gram-negative bacteria such as *Staphylococcus aureus* and *Escherichia coli* [20,21]. Mg (OH)_2_ also causes direct membrane damage upon contact and has shown efficacy against sulfate-reducing and other pathogenic bacteria [22]. Magnesium hydride (MgH_2_), although less studied, has recently attracted attention for its hydrogen-releasing capability, which generates an alkaline microenvironment that disrupts biofilms and suppresses oral pathogens such as *Streptococcus mutans* and *Porphyromonas gingivalis* [23]. Despite these promising properties, the application of Mg-based nanocoatings on textile substrates remains underexplored.

Metal nanoparticles have an antialgal effect on microalgae of the *Prototheca genus*. Silver nanoparticles’ action on *Prototheca* spp. was demonstrated by a few studies performed by Montrenko et al. [24], Jagielski et al. [25] and others. However, there is no direct study on the effect of magnesium (Mg) nanoparticles against this microalga.

Although some studies have incorporated MgO nanoparticles into polymer films and nanofiber mats for antimicrobial wound dressings [26], few have investigated Mg-coated woven or nonwoven textiles, particularly their effects on endothelial cells such as HUVECs.

Magnesium compounds can be used to coat textiles to improve their properties. Magnetron sputtering enables the deposition of nanostructured coatings onto textile surfaces, enhancing their functional properties such as antibacterial activity, ultraviolet (UV) protection and hydrophobicity. Magnetron sputtering is a well-established and widely accepted technique, valued for its effectiveness in both small-scale scientific studies and large-scale industrial manufacturing. Compared to other synthesis methods, it is particularly recognized for its scalability, adaptability, consistent film quality and high reproducibility [27]. Operating under vacuum conditions, this method generates a plasma that ejects atoms from a solid target material, which are then deposited onto a substrate—in this case, textile fibers. While the direct sputtering of magnesium (Mg) onto textiles remains underexplored, related studies highlight the potential of magnesium-based coatings in biomedical applications. For example, Mg coatings sprayed onto metal substrates have demonstrated both antibacterial properties and cytocompatibility [28], while MgO particles embedded in textile polymers provided notable antimicrobial activity and surface functionality improvements [29].

One study demonstrated the fabrication of nano-sculpted Mg thin films using magnetron sputtering combined with glancing angle deposition, allowing for controlled surface microstructures by tuning the deposition parameters—an approach that could be translated to textile substrates [30]. In parallel, research on magnesium-containing compounds, such as brucite (Mg(OH)_2_), applied to textiles has shown effective antibacterial activity against pathogens, including *Escherichia coli* and *Staphylococcus aureus* [31]. Although Mg is less often used compared to common metals such as silver or copper, these results support the adaptability of deposition strategies and functional benefits for Mg-based systems [32].

To date, however, no studies have systematically investigated the dual performance of plasma-sputtered Mg coatings on cotton textiles—namely, their antimicrobial and antialgal efficacy alongside their cytocompatibility with endothelial cells. In this study, we address this gap by employing low-temperature magnetron sputtering, a clean and solvent-free technique, to deposit Mg-based nanoparticles onto medical-grade cotton fabrics, aiming to develop multifunctional coatings that support both microbial and algal inhibition and cellular viability.

## 2. Results

### 2.1. X-Ray Photoelectron Spectroscopy (XPS) Analysis

**Figure 1:**
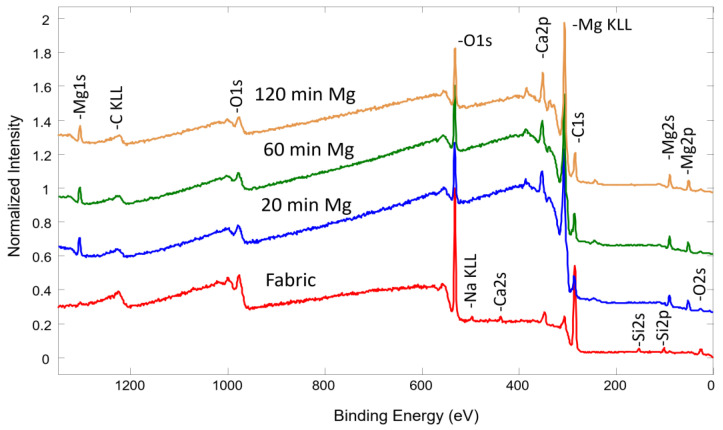
XPS survey spectra of pure fabric and synthesized with Mg using different deposition times.

The analysis of the elemental concentration in the top layer of the deposited thin film was performed using the X-ray Photoelectron Spectroscopy (XPS) technique. Figure 1 illustrates the obtained spectra at different Mg deposition conditions, while Table 1 presents the calculated elemental concentration.

The control textile fabric primarily consisted of carbon (C) and oxygen (O), along with small amounts of calcium (Ca), silicon (Si) and potassium (K). As expected, the Mg concentration exhibited a progressive increase with longer deposition durations, reaching 18.1 at. %, 19.8 at. % and 21.3 at. % after 20 min, 60 min and 120 min of synthesis, respectively. Simultaneously, the carbon concentration exhibited a significant reduction, decreasing from approximately 61 at. % to 33 at. %, as it is an outcome of Mg deposition. Meanwhile, the oxygen concentration increased slightly, likely due to the formation of new Mg–O-based chemical bonds, indicating possible oxidation or chemical interaction between the deposited Mg and the fabric’s surface.

**Figure 2:**
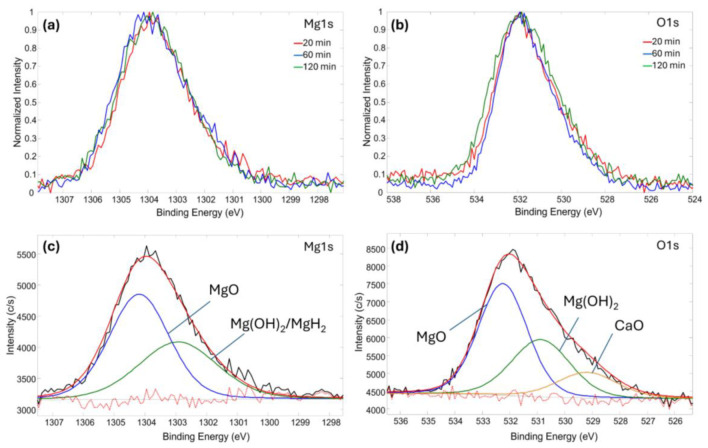
XPS fitting results of Mg-based thin film deposited on medical fabric: (**a**) Mg 1s spectra of all deposited samples; (**b**) O 1s spectra of all deposited samples; (**c**) Mg 1s spectra deconvolution after 20 min deposition time; (**d**) O 1s spectra deconvolution after 20 min deposition time. In (**c**,**d**): red dotted line—background, black line—as measured curve, red line—deconvoluted curve.

The chemical bond analysis of the Mg-based thin films deposited on medical fabric was performed using the XPS technique. Figure 2a,b display spectra with highly similar shapes, representing the samples synthesized at different deposition times. A consistent trend was observed across both Mg 1s and O 1s spectra, indicating that the chemical structure of the deposited films remained stable regardless of the deposition duration. A more detailed analysis was conducted for the sample synthesized for 20 min.

The results of the high-resolution Mg 1s spectral deconvolution revealed the presence of two distinct chemical states: MgO at 1304.2 eV and a combined contribution from Mg(OH)_2_ and MgH_2_ at 1302.9 eV. Due to the minimal binding energy difference (~0.1 eV) between Mg(OH)_2_ and MgH_2_, a direct separation of these components is challenging. However, a quantitative analysis of the Mg 1s peak areas indicated an intensity ratio of approximately 1:1.5 between the Mg(OH)_2_/MgH_2_ component and MgO, implying that MgO is the predominant magnesium-containing species in the uppermost surface layer. In parallel, the O 1s spectrum exhibited three distinct components: CaO at 529.2 eV, Mg(OH)_2_ at 530.9 eV, and MgO at 532.2 eV. The integrated area ratio of Mg(OH)_2_ to MgO from the O 1s spectrum was approximately 1:1.8, which differs from the ratio obtained in the Mg 1s region. This discrepancy suggests that not all of the low binding energy contribution in the Mg 1s spectrum can be attributed solely to Mg(OH)_2_. Considering the chemical plausibility and the absence of a corresponding oxygen contribution, the excess signal in the Mg(OH)_2_/MgH_2_ region is reasonably assigned to MgH_2_. Therefore, the combined analysis of both the Mg 1s and O 1s spectra supports the presence of MgH_2_ as a distinct chemical phase within the near-surface region of the deposited film.

In addition to the surface chemical analysis, a coating thickness evaluation was conducted. The film thickness measurements were attempted using a profilometer on the Mg-based nanoparticles deposited for 120 min under identical conditions onto a quartz substrate. However, the measured values were below the instrument’s reliable detection threshold, precluding an accurate quantification. Thickness estimates were therefore derived from the XPS data, which indicated a Mg content of 21.3 at. % after 120 min of deposition. Considering the XPS information depth of approximately 8–10 nm, the corresponding Mg-based nanocluster layer thickness was estimated to be 1.7–2.1 nm. By extrapolation, the thicknesses for the 60 min and 20 min depositions were calculated to be approximately 1.5–2.0 nm and 1.4–1.8 nm, respectively.

### 2.2. SEM Analysis

**Figure 3:**
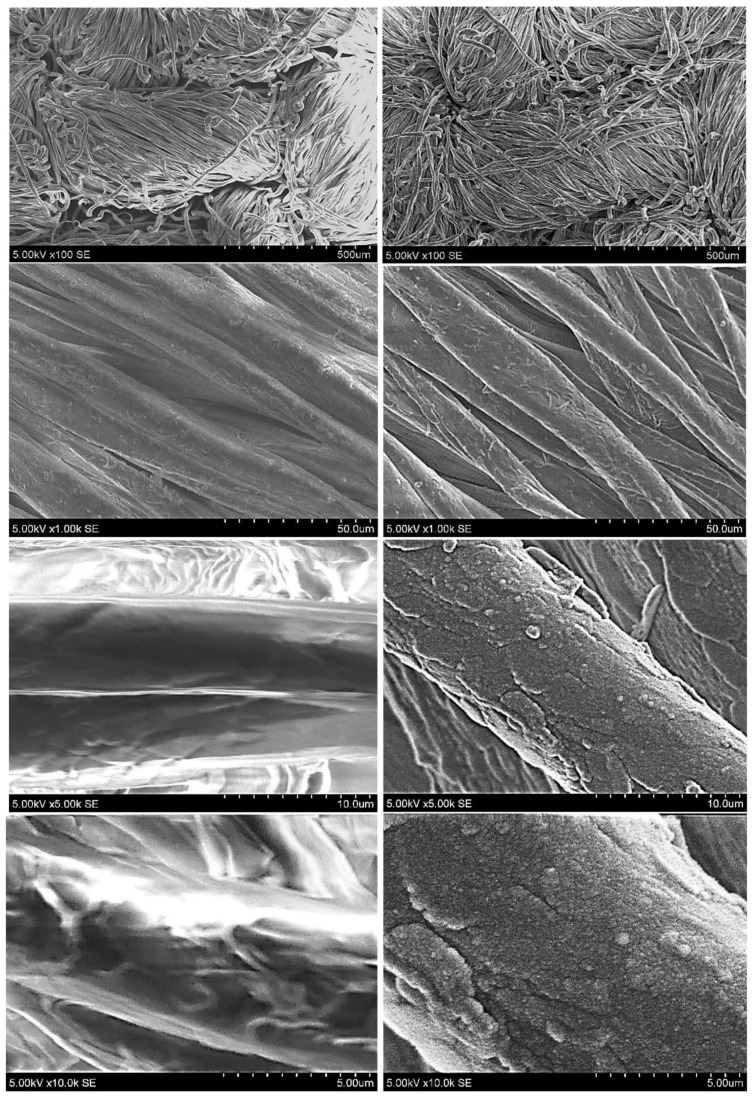
SEM images of cotton textile before (**left panel**) and after (**right panel**) Mg-based nanoparticle deposition at 350 W for 60 min, shown at magnifications of 100×, 1000×, 5000× and 10,000×. The scale bars are expressed in micrometers (μm).

The surface morphology was examined with a Hitachi S-3400 N scanning electron microscope (Hitachi, Tokyo, Japan) equipped with a secondary electron detector. The samples were assessed under low vacuum conditions with a beam energy of 5 kV, using magnifications of 100×, 1000×, 5000× and 10,000×. Figure 3 presents the SEM images of the samples before and after plasma treatment, highlighting the differences induced by the plasma sputtering process.

The SEM analysis revealed distinct differences between the control textile and the Mg-coated textile after plasma sputtering. The untreated textile at 5000× magnification showed separated fibers with a relatively smooth surface and no coating. At higher magnifications, no significant surface modifications were observed (Figure 3). In contrast, after 1 h of plasma sputtering with magnesium, the morphology of the textile changed significantly. The Mg-coated sample exhibited generally consistent particle coverage with some localized accumulation on certain fiber regions, accompanied by an increased surface roughness, indicating successful Mg deposition. The coated textile displayed a roughened layer with the magnesium particles adhering to the fibers, and at 10,000× magnification, interconnected particle clusters were clearly visible. These localized accumulations were more pronounced in areas with a higher surface roughness or at fiber intersections and bends, reflecting the influence of the underlying textile structure on the coating distribution.

Previous research suggests that Mg-based coatings enhance material properties such as hydrophobicity and potential hydrogen storage capabilities [33]. Plasma sputtering typically results in a conformal coating, with deposition rates and surface roughness varying according to the sputtering duration and plasma parameters [34]. Magnesium coatings have also been shown to influence mechanical strength and chemical reactivity [35,36].

**Figure 4:**
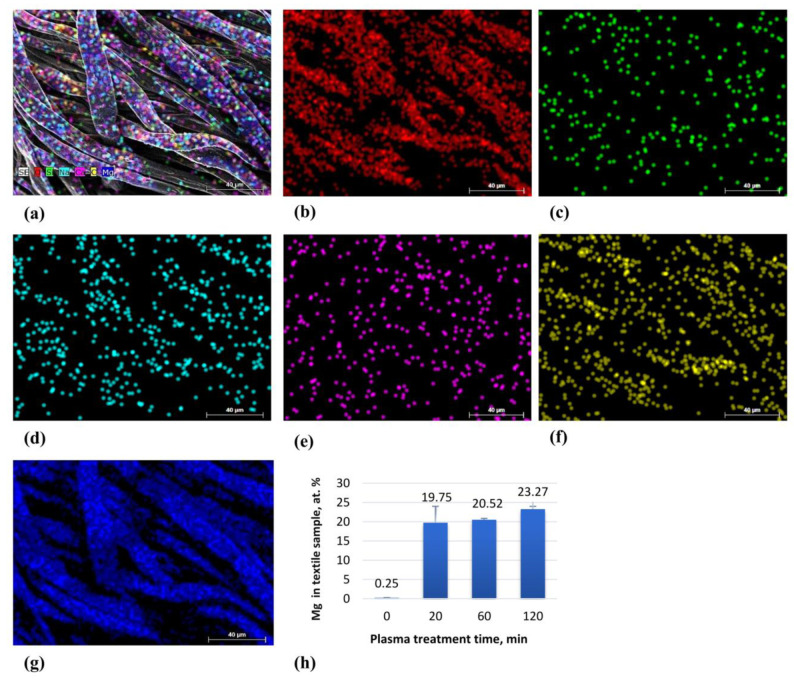
The elemental mapping view of all elements together (**a**) and deposited separately on textile surface: element O (**b**), element Si (**c**), element Na (**d**), element Ca (**e**), element C (**f**), element Mg (**g**) and EDS of the element Mg in the textile sample (**h**).

To assess the elemental composition and spatial distribution of the coating, EDS mapping was conducted on selected areas observed under SEM (Figure 4). The analysis confirmed a generally consistent distribution of Mg and O across the surfaces, with minor variations between regions. Additional elements—including carbon (C), calcium (Ca), sodium (Na) and silicon (Si)—were also identified, corresponding to the inherent composition of the cotton substrate.

### 2.3. Antimicrobial and Antialgal Activity Analysis

**Figure 5:**
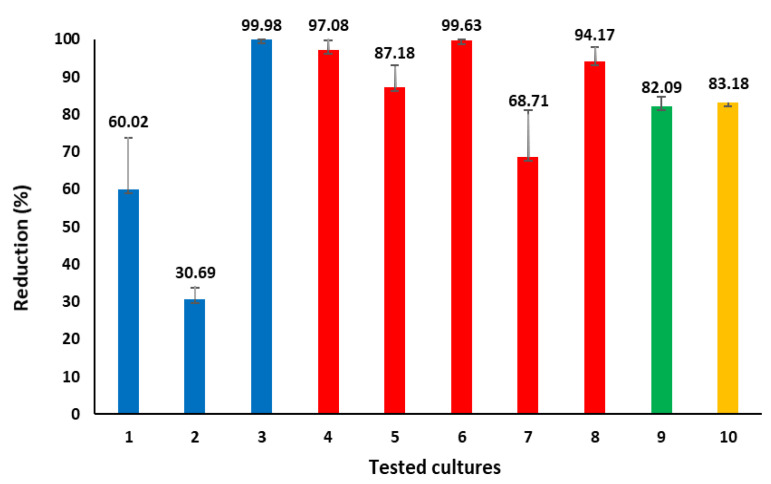
The antimicrobial and algal activity of Mg nanoparticle-coated textiles against 10 microorganisms. Growth reduction in the percentage of microorganisms and microalgae compared to the control material (W = 350 W, t = 120 min) is shown. Error bars represent the standard deviation between three separate iterations. The red bars represent Gram-negative bacteria; the blue bars, Gram-positive bacteria; the green bar, *Candida albicans* fungus; and the orange bar, microalgae *Prototheca* spp. 1—MRSA *Staphylococcus aureus*, 2—*Enterococcus faecalis*, 3—*Bacillus cereus* REF11778, 4—*Pseudomonas aeruginosa* REF27853, 5—*Klebsiella pneumoniae* ATCC 10031, 6—*Acinetobacter baumannii*, 7—*Escherichia coli* REF 25922, 8—*Salmonella enterica*, 9—*Candida albicans* REF10231, 10—*Prototheca* spp.

The antibacterial and antialgal effectiveness of the Mg-coated textile was assessed against 10 strains, including 8 bacterial strains (both Gram-positive and Gram-negative), 1 fungal strain (*Candida albicans*) and 1 algal strain (microalgae *Prototheca* spp.) of reference, clinical and zoonotic origins. The growth reduction, described in the corresponding percentage following exposure to the coated textile, in all cases, except for *Enterococcus faecalis,* was over 60%; for four bacterial strains, it resulted in almost total growth reduction (*Bacillus cereus* REF11778, *Pseudomonas aeruginosa* REF27853*, Acinetobacter baumannii* and *Salmonella enterica*) (Figure 5). The inhibition for *C. albicans* and *Prototheca* spp. was 82% and 83%, respectively.

### 2.4. Effects of Mg-Coated Textile Extracts on Mammalian Cell Viability

**Figure 6:**
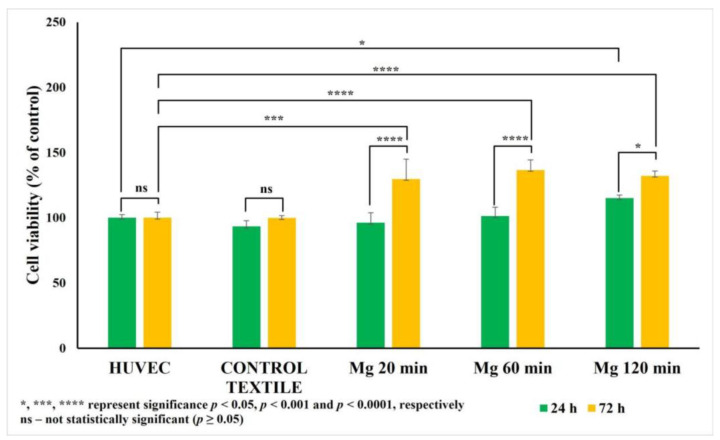
The effect of Mg-coated textiles on HUVEC viability assessed by MTT assay. HUVECs were cultured in the presence of Mg-coated textile extracts (Mg20, Mg60, Mg120) or extracts from uncoated textiles for 24 and 72 h. Results are expressed as mean ± SD from three independent experiments, each performed in triplicate. A statistically significant increase in viability was observed after 72 h for Mg20 and Mg60 groups (*p*  <  0.05), compared to control (untreated HUVECs).

The MTT assay revealed a significant increase in cell viability after 72 h of incubation with Mg-coated textiles, particularly in the Mg20 and Mg60 groups, compared to both the uncoated textile and untreated control (HUVECs). This suggests a possible stimulatory effect of Mg coatings on cell proliferation or metabolic activity (Figure 6).

The Mg120 group maintained viability levels similar to the control, indicating no cytotoxicity even at higher deposition times. These findings align with the known effects of Mg^2+^ ions on endothelial cells, which respond positively to physiological and moderately elevated Mg^2+^ levels by increasing proliferation and metabolic activity [37].

**Figure 7:**
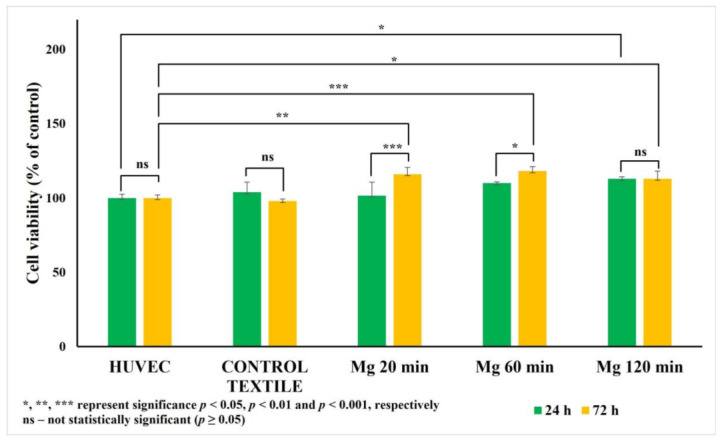
The effect of Mg-coated textiles on HUVEC viability assessed by PrestoBlue assay.

Importantly, the PrestoBlue assay also showed a consistent increase in metabolic activity, especially in the Mg20 and Mg60 groups after 72 h. This confirms the results observed in the MTT assay and further supports the hypothesis that the compound has a proliferative and cytoprotective effect. Unlike the MTT assay, which is limited to mitochondrial reduction capacity, the PrestoBlue assay reflects the broader redox state of the cell, and its agreement with the MTT assay indicates a genuine improvement in cell viability rather than a stress response or metabolic imbalance (Figure 7).

Taken together, the results from both the assays indicate that the Mg-based nanoparticles coated textiles do not exert cytotoxic effects on HUVECs. On the contrary, they may even promote cell viability and proliferation over time, highlighting their potential biocompatibility for biomedical or wound-healing applications.

## 3. Discussion

The increasing demand for antimicrobial and biocompatible materials in biomedical applications—particularly for wound care, vascular grafts and implantable textiles, as well as antialgal applications to control and eliminate algae, often in aquatic environments—has driven extensive research into surface modification strategies. Although nanocoatings with silver or copper nanoparticles are well-established for their antimicrobial activity, their clinical use is increasingly limited by concerns regarding cytotoxicity, bioaccumulation and environmental persistence [38]. This presents a critical challenge: how to develop surface modifications that inhibit microbial or algal growth while supporting host cell function.

Magnesium, as a physiologically essential element with well-documented roles in cellular health, offers an attractive alternative to traditional metallic biocides [39,40,41]. A controlled presence of Mg at the surface can provide antimicrobial and antialgal protection while maintaining cytocompatibility, making it well-suited for tissue-contacting applications. This dual functionality was supported by our biological evaluation using HUVECs, which demonstrated that Mg-coated textiles were not only non-toxic but also enhanced cell viability, particularly in the samples sputtered for 20 and 60 min. Such proliferation-supporting effects are likely driven by the local release of Mg^2+^ ions into the surrounding microenvironment [36]. Elevated concentrations of Mg^2+^ (up to 5.0 mM) have previously been shown to stimulate endothelial cell proliferation and migration, while a deficiency impairs these functions and increases oxidative stress [16,18,19]. Our findings are consistent with this evidence and further reinforce the view that surfaces incorporating magnesium exhibit active biofunctionality rather than being merely inert.

In parallel, the antimicrobial testing of eight bacterial strains revealed that the Mg-based nanoparticles (NPs) were able to inhibit the growth of Gram-negative bacteria by 90% or more, except *Escherichia coli* REF 25922 (less than 70%). Gram-positive bacteria exposed to cotton coated with Mg-based nanoparticles were inhibited less effectively, by 30 to 99 percent (the smallest inhibition was observed for *E. faecalis* and the highest was for *B. cereus*). It confirms, except for Gram-positive bacteria *Bacillus cereus* REF11778 (in our study), the results of Nguyen et al.’s study that MgO NPs eliminate Gram-negative bacteria more effectively than Gram-positive bacteria, possibly due to differences in the structures of their bacterial cell walls and membranes [20]. The inhibition of Gram-positive bacteria was diverse in percentage in our study. The antibacterial activity of the metal/metal oxide, including Mg NPs, depends on the metal/metal oxide type, nanoparticle size, morphology, dose, etc., as well as on the *species of microorganism* [42]. It is known that the Gram-positive bacteria *E. faecalis* might survive in an unsupportive environment, possibly due to several factors: (i) its tolerance to alkaline stress [43], (ii) its survival by entering a “viable but non-culturable state” [44] and (iii) its ability to form biofilms [45,46]. A possible additional explanation for the different bacterial susceptibility to Mg-based nanoparticles is a modulation of the magnesium ion flux. Lee and colleagues detected two cell types within the same population. Some cells experienced sudden changes in membrane potential because they poorly regulate the magnesium ion flux through the membrane, making them more vulnerable to MgNPs, and they died. When other cells managed to modulate the magnesium ion flux and keep their membrane potential stable under unfavorable conditions, they could survive [47]. Contact between Mg NPs and microorganisms’ cell membrane induces oxidative stress, damages cell membrane integrity, increases cell permeability, affects the synthesis of intracellular proteins, etc. [22,48]. It is worth highlighting that Mg NP coatings achieved effective microbial suppression without compromising host cell viability. This trade-off is especially advantageous in applications where minimizing cytotoxicity is critical [24]. The contrasting response between the pathogen cells and the human endothelial cells can be attributed to fundamental differences in cell structure and physiology: in pathogens, Mg-based coatings trigger lethal effects through membrane damage and related biochemical stress, while in endothelial cells, a controlled Mg^2+^ ion presence supports essential metabolic and structural functions, thereby promoting viability and proliferation rather than cytotoxicity. The antimicrobial activity of Mg and Mg compounds such as MgO has been attributed to ROS generation, membrane disruption and pH elevation [48,49]. Additionally, MgH_2_ may exert antimicrobial effects via hydrogen release in aqueous solutions, creating reductive environments that destabilize microbial biofilms [23]. Emerging evidence also suggests that Mg^2+^ may influence microbial community structure systemically, potentially offering regulatory benefits beyond local antimicrobial effects [50]. The activity of metal nanoparticles against microalgae *Prototheca* spp. of clinical or bovine origin has previously been tested using silver nanoparticles in a few studies [51,52]. However, the antialgal activity of Mg-based nanoparticles has been tested for the first time in this study. The results are promising, as the effect of Mg decreased the growth of *Prototheca* spp. by 83%. *Prototheca*-specific studies are limited; thus, the response to Mg NPs might be explained by findings on fungal-like eukaryotes such as *C. albicans*, whose response to the Mg NPs was 82%. Kong et al. demonstrated that MgO NPs inhibit growth, initial adhesion, two-phase morphological transformation and biofilm formation in a time- and concentration-dependent manner [53]. MgO NPs provoke a synergistic fungal cellular stress response, as they engage with fungal cell membranes through electrostatic interactions, which leads to the disruption of both the membranes and the glucan matrix. The heightened oxidative stress, the release of Mg^2+^ and the DNA damage, subsequently impeding protein synthesis, disrupting proteins and leading to intracellular leakage, is followed by cell death [54,55].

A key aspect of harnessing Mg’s functional potential lies in its delivery method. In this study, we utilized magnetron sputtering—a solvent-free, controllable and scalable technique—to deposit Mg-based nanoparticles onto medical-grade cotton textiles. Plasma sputtering allows the precise adjustment of the film thickness, elemental composition and surface morphology even on porous or heat-sensitive substrates, making it particularly suitable for biomedical textiles [56,57].

The surface characterization performed confirmed the successful deposition of Mg coatings on cotton textiles, as evidenced by the compositional and morphological analyses. The SEM imaging revealed a generally consistent nanoparticle distribution and increased fiber roughness, features often correlated with improved hydrophilicity and enhanced cellular adhesion [37], while the EDS mapping confirmed the Mg and O distribution across the fabric surface and the XPS analysis identified bioactive Mg, MgO and Mg (OH)_2_/MgH_2_ phases. The longer sputtering times correlated with an increased Mg signal intensity, confirming deposition control. Notably, trace amounts of CaO were detected in all the samples, including the untreated controls, raising questions about the influence of manufacturing additives. Calcium compounds such as CaO are frequently used in medical textiles for pH buffering and antimicrobial enhancement [58]. Calcium salts are also important in enzyme-based cotton desizing, as calcium ions stabilize the alpha-amylase enzymes that hydrolyze starch into soluble fragments [59]. However, in this study, the Ca levels remained unchanged across the treated and untreated samples, indicating that the observed biological effects were primarily driven by the Mg coating rather than pre-existing additives. This distinction is crucial for understanding causality in bio-interface performance.

These findings collectively validate the concept of magnetron-sputtered Mg coatings as dual-functional systems that offer both cytoprotection and control of microbial and algal growth. The technique’s scalability, environmental compatibility and material precision make it highly suitable for integration into biomedical manufacturing pipelines. The potential applications include wound dressings, vascular grafts, implantable meshes and tissue-engineering scaffolds. Beyond the medical field, Mg-based coatings may also be relevant in biodegradable food packaging, agricultural films and smart sensor platforms where antimicrobial and antialgal activity and safety are equally important [60,61,62].

## 4. Materials and Methods

### 4.1. In Situ Formation of Mg-Based Nanoparticles on Textile Substrates via Magnetron Sputtering

The experimental textile material was made of 100% cotton fabric with a density of 138 gsm. Before use, the untreated cotton fabric (used as a control sample) was sterilized in an autoclave. The fabric was precisely cut into 10 mm × 10 mm squares using sterile scissors and placed into sample holders. Before depositing Mg-based nanoparticles using low-temperature plasma, the sterility of all cotton fiber samples was verified using thioglycolate medium (Thermo Fisher Scientific, Basingstoke, UK).

**Figure 8:**
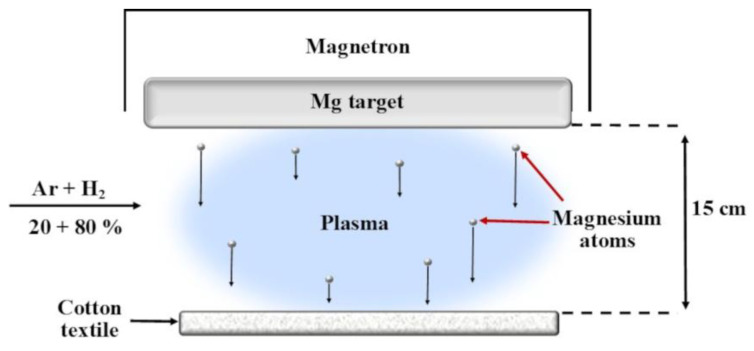
Experimental setup for the deposition of Mg-based nanoparticles on the textile material.

Magnetron sputtering was applied to deposit Mg-based nanoparticles on the textile material. A DC power supply (Advanced Energy Sparc-Le 20, Fort Collins, CO, USA) was used. The sputtering target was a magnesium cathode with 99.99% purity, 10 mm in diameter and 2.5 mm in thickness, supplied by Kurt J. Lesker. The distance between the target and the textile sample was 15 cm. The distance magnetron–textile target was chosen to keep textile temperature lower than 100 °C, as is usually done during disinfection procedures. Cotton remains intact mechanically and chemically at these temperatures. Before the actual deposition, the magnesium target underwent a pre-sputtering process for approximately 10 min. A schematic illustration of the magnesium particle deposition using magnetron sputtering is shown in Figure 8.

The textile sample was placed inside a vacuum chamber beneath the Mg magnetron. Plasma was generated using DC power sources, with an argon–hydrogen mixture as the primary working gas. The mixture ratio was 20–80% (Ar–H_2_). Due to hydrogen’s low atomic mass, sputtering predominantly produces atomic species from the cathode, thereby minimizing the formation of larger clusters typically observed with heavier gases such as argon. The basic configuration of the setup, including the positioning of the electrodes and the textile, ensured a generally consistent coating with the selected Mg nanoparticle–plasma material, as shown in Figure 1.

Low-temperature plasma was generated using DC power at 350 W. The system reached a base vacuum of 0.04 Pa, and the deposition pressure was consistently maintained at 10 Pa throughout the process. The deposition was conducted for 20, 60 and 120 min.

### 4.2. Characterization of Materials

Various characterization techniques were employed to evaluate the properties of the textile samples. Surface morphology was examined using a scanning electron microscope (SEM, Hitachi S-3400 N, Tokyo, Japan) equipped with a secondary electron detector. Topographical analysis was performed under low vacuum conditions at a beam energy of 5 kV, with images captured at magnifications of 100×, 1000×, 5000× and 10,000×. Elemental distribution within the samples was assessed using energy-dispersive X-ray spectroscopy (EDS, Bruker Quad 5040, Billerica, MA, USA). X-ray Photoelectron Spectroscopy (XPS) measurements were carried out using a PHI Versaprobe spectrometer equipped with an Al monochromator (25 W beam power, 100 µm beam size). The energy scale was calibrated based on the Au 4f7/2 (84.0 eV) and Cu 2p3/2 (932.6 eV) reference peaks. To prevent surface charging during analysis, a dual charge neutralization system—comprising low-energy electron and ion sources—was used, and the effect of charging was corrected by referencing the adventitious carbon C1s peak to 284.8 eV. Spectral deconvolution was performed using a consistent fitting procedure with a Shirley background and Gaussian–Lorentzian peak shapes. To analyze the elemental composition of the top layer of textiles with Mg-based nanoclusters, three XPS measurements were performed on each sample type (pristine fabric and samples with 20, 60 and 120 min of Mg deposition). The results were averaged, and the mean values are reported in Table 1. The relative accuracy of the atomic concentration measurements is estimated to be less than 3% for major elements, while for trace element (Na), higher uncertainty is expected due to signal-to-noise limitations and matrix effects.

### 4.3. Evaluation of Antimicrobial and Antialgal Activity

The antibacterial and antialgal properties of cotton fabric samples—coated on one side with Mg-based nanoparticles using 350 W DC power for deposition durations of 120 min—were evaluated against reference, clinical and zoonotic microorganisms and zoonotic microalgae.

A total of 10 strains, including both Gram-negative and Gram-positive bacteria as well as yeasts and microalgae, were obtained from the microbial culture collection of the Institute of Microbiology and Virology at the Lithuanian University of Health Sciences. The antimicrobial activity of the Mg nanomaterial was assessed using the same methodology described in our previously published work [63]. A microalgae strain was screened using the Kirby–Bauer technique on Mueller–Hinton agar plates to evaluate *Prototheca* spp. susceptibility to antifungal medications. It has resistance to antifungals amphotericin B (4 µg/mL), flucytosine (35 µg/mL), econazole (16 µg/mL), miconazole (16 µg/mL) and fluconazole (16 µg/mL).

The antialgal activity of cotton coated with Mg-based nanoparticles was performed as follows. A suspension of *Prototheca* spp. was prepared in a physiological solution with a density of 0.5 according to the McFarland standard. A 1 × 1 cm piece of the test material was placed in a sterile Petri dish. An amount of 50 µL of microalgal suspension was loaded onto it. The plates were covered and kept at room temperature and under laboratory lighting conditions for 1 h.

Additionally, a physiological solution was prepared for 10-fold dilutions of bacterial culture—the first tube with 10 mL of physiological solution, the rest of them with 9 mL of physiological solution. Squares were kept for an hour in Petri dishes, and then they were transferred into a tube with 10 mL of physiological solution. The tube was vortexed, and then 10 mL of liquid was transferred to a tube with 9 mL of physiological solution. The same action was repeated three times. The 10-fold dilutions were from 10^1^ to 10^−4^. A total of 50 µL of liquid from ten-fold dilutions of the samples were plated on soya agar (three plates for each dilution). From each test tube, 50 µL was inoculated onto Sabouraud Dextrose agar, three plates for each dilution. The plates were cultured in a thermoregulated growth chamber at 30 °C for 24 h. The average of three plates was calculated. The percentage reduction across all ten strains was determined relative to the control. A sterile piece of cotton textile was taken for control.

### 4.4. Cultivation of Mammalian Cells

The human umbilical vein endothelial cell (HUVEC) line was sourced from Gibco (Gibco by Life Technologies, Carlsbad, CA, USA). Cells were cultured in 25 cm^2^ cell culture flasks (Nunclon Delta Surface, Thermo Fisher Scientific, Waltham, MA, USA) using DMEM/F12 medium (Gibco by Life Technologies, Carlsbad, CA, USA), supplemented with Large Vessel Endothelial Supplement (LVES 50X, Gibco by Life Technologies, Carlsbad, CA, USA) and a penicillin/streptomycin antibiotic solution (Gibco by Life Technologies, Carlsbad, CA, USA). Cultures were maintained in a humidified cell incubator (HERA Cell 150i, Thermo Fisher Scientific, Waltham, MA, USA) at 37 °C with 5% CO_2_. Cell morphology was monitored daily using an EVOS XL Core inverted microscope (Invitrogen Co., Carlsbad, CA, USA). After 2–3 passages, the cells entered the logarithmic growth phase. Trypsinization was performed using 0.05% trypsin–EDTA solution (Gibco by Life Technologies, Carlsbad, CA, USA) for 1–2 min at 37 °C. The cells were then stained with 0.4% trypan blue (Gibco by Life Technologies, Carlsbad, CA, USA) and counted using a hemocytometer (Thermo Fisher Scientific, Waltham, MA, USA).

### 4.5. Cell Viability Assays

Two complementary methods were employed to evaluate the cytotoxic effects of Mg extracts on cell cultures, following the recommendation to use multiple assays for reliable assessment of cell viability [64]. The selected techniques included the PrestoBlue assay, which detects overall metabolic activity, and the MTT assay, which specifically measures mitochondrial enzyme function [65].

Both MTT and PrestoBlue assays are widely used for in vitro cytotoxicity and viability testing, as they assess cellular metabolic activity through different mechanisms [66,67]. The MTT assay is considered a benchmark method due to its reliability, whereas the PrestoBlue assay offers higher sensitivity and allows for rapid, real-time analysis. The combined use of both assays provides a more comprehensive evaluation of cellular responses to the tested materials [66]. In this study, they were jointly applied to robustly assess the cytotoxic potential of Mg-coated textiles, ensuring a thorough analysis of cell viability and metabolic function [68].

For extract preparation, Mg-coated textile samples were cut into 1 × 1 cm^2^ squares, placed into 24-well culture plates and immersed in 1 mL of DMEM/F12 medium per well. Three replicates were prepared for each sample type. The plates were incubated for 24 h at 37 °C in a humidified atmosphere containing 5% CO_2_. Control samples were incubated under identical conditions. After incubation, the resulting Mg extracts were collected and transferred into sterile microtubes for further cytotoxicity analysis.

#### 4.5.1. MTT Assay—Evaluation of Mitochondrial Enzyme Activity

The MTT assay was used to evaluate the mitochondrial metabolic activity of endothelial cells exposed to Mg-coated textile extracts. This colorimetric assay relies on the enzymatic reduction of the yellow tetrazolium salt MTT to insoluble purple formazan crystals by mitochondrial succinate dehydrogenases in viable cells [65]. HUVECs were seeded at 5 × 10^3^ cells per well in 96-well plates and incubated for 24 h to ensure proper attachment. The cells were then treated with textile extracts and incubated for an additional 24 or 72 h.

Following treatment, the medium was replaced with MTT reagent (5 mg/mL in PBS) (Invitrogen Co., Carlsbad, CA, USA), and the cells were incubated for 2 h at 37 °C. Formazan crystals formed in metabolically active cells were dissolved using DMSO solution, and absorbance was measured at 570 nm in a plate reader (TECAN INFINITE M PLEX, Tecan Group Ltd., Männedorf, Switzerland). The results were expressed as a percentage of viable cells relative to the untreated control.

#### 4.5.2. PrestoBlue Assay—Real-Time Measurement of Metabolic Viability

To complement the MTT assay and enable real-time, non-destructive assessment of cellular metabolic activity, the PrestoBlue assay was applied. This resazurin-based reagent undergoes a reduction to resorufin by mitochondrial and cytosolic enzymes in metabolically active cells, resulting in a measurable colorimetric and fluorometric change. Unlike the MTT assay, the Presto Blue assay does not require solubilization steps and enables live-cell monitoring.

After 24 h and 72 h incubation with Mg-coated textile extracts, culture media were removed and replaced with fresh media containing diluted Presto Blue reagent. Plates were incubated for 20 min at 37 °C. Fluorescence was detected at 570 nm (excitation) and 610 nm (emission), and absorbance readings were also taken at 570 nm. Viability was calculated relative to untreated control wells.

### 4.6. Statistical Analysis

Cell viability was assessed in 3 to 4 replicates, with each sample measured in duplicate. The outcomes were compared against a control group, and statistical tools were employed to validate the reliability of the results. Statistical analysis was conducted using GraphPad Prism version 10.4.1. Data in the figures are presented as the mean ± standard error of the mean (SEM). To assess statistical significance, a two-way ANOVA was followed by Tukey’s post hoc test. Results were deemed statistically significant at *p* < 0.05.

Antimicrobial activity data were analyzed using R statistical software (version 3.6.2, R-project.org). A significant threshold of *p* < 0.05 was also applied here. Microsoft Excel for Microsoft 365 MSO (Version 2312, Build 16.0.17126.20132) 64-bit was used to generate graphical representations.

## 5. Conclusions

Our study presents a novel approach to developing multifunctional textile coatings by combining magnesium’s biological benefits with the advantages of magnetron sputtering. These bioactive surfaces offer a sustainable and effective solution for balancing infection control of clinical and zoonotic origins and tissue compatibility, laying the foundation for next-generation medical and bio-interactive materials.

The synthesized coatings consisted primarily of Mg, MgO and Mg(OH)_2_/MgH_2_ phases, with the XPS analysis confirming the dominant presence of MgO and the contributions from MgH_2_ at the surface. The SEM and EDS analyses revealed a generally consistent Mg distribution and an increased surface roughness, with the Mg content reaching up to 21.3% after 120 min of deposition.

The coatings demonstrated effective antibacterial activity against both Gram-positive and Gram-negative bacteria—except *Enterococcus faecalis.* Additionally, antifungal efficacy against *Candida albicans* and a strong antialgal effect against *Prototheca* spp.—with over 80% growth reduction after the exposure to cotton fabric samples that were coated on one side with Mg-based nanoparticles using 350 W DC power for deposition durations of 120 min—were achieved, marking the first reported inhibition of this microalga by Mg-based nanoparticles.

The in vitro cytocompatibility tests using HUVECs confirmed the absence of cytotoxicity for all the tested coatings. Notably, both the MTT and PrestoBlue assays showed increased metabolic activity after 72 h, particularly for the 20 and 60 min coatings, indicating a cytoprotective and proliferation-promoting effect likely mediated by a controlled Mg^2+^ ion presence. The 120 min coatings combined these biocompatibility attributes with the highest antimicrobial and antialgal performance, making them the optimal condition for achieving both infection control and host cell safety.

These findings support plasma-sputtered Mg coatings as a dual-functional platform, providing both antimicrobial and antialgal protection while supporting endothelial cell compatibility. Future studies should explore long-term cellular responses, biological performance under physiologically relevant conditions and the development of hybrid coatings to further enhance functionality. Overall, this work highlights magnesium’s potential as a sustainable coating material and magnetron sputtering as a versatile technique for engineering next-generation biomedical textiles.

## Figures and Tables

**Table 1:** XPS elemental concentration of medical fabric with deposited Mg films (averaged mean values and scatter).

Sample	Concentration, at. % (Mean ± SD *)					
	C	O	Ca	Si	Na	Mg
Control textile	61.2 ± 1.6	34.5 ± 0.5	3.2 ± 0.4	1.6 ± 0.2	0.2 ± 0.1	-
20 min	33.6 ± 0.7	38.7 ± 0.6	8.3 ± 0.3	1.2 ± 0.0	0.1 ± 0.0	18.1 ± 0.5
60 min	34.7 ± 0.4	36.5 ± 0.2	7.6 ± 0.1	1.3 ± 0.2	0.2 ± 0.1	19.8 ± 0.6
120 min	33 ± 0.5	37.4 ± 0.6	7.2 ± 0.5	1.1 ± 0.3	0.1 ± 0.0	21.3 ± 0.5

* SD—standard deviation.

## Data Availability

Data are contained within the article.
